# Supplementary material for: Mindboggling morphometry of human brains
Source: PLoS Comput Biol. 2017 Feb 23;13(2):e1005350. doi: 10.1371/journal.pcbi.1005350 (PMC5322885; doi:10.1371/journal.pcbi.1005350)
Supplement: S1 Supplement — (PDF) [file pcbi.1005350.s001.pdf]

S1 Appendix: Mindboggle output directory tree

Mindboggle supplement (http://mindboggle.info)

This directory tree shows outputs from most, but not all, optional arguments, and does not include interim results stored in a working directory or downloaded files in a cache directory:

Topmost directory of Mindboggle output for subject $SUBJECT:
mindboggled/$SUBJECT/

Cortical surfaces with folds, sulci, fundi, or in MNI152 space:
features/[left,right]_cortical_surface/
- folds.vtk
- sulci.vtk
- fundus_per_sulcus.vtk
- cortex_in_MNI152_space.vtk

Cortical surface labels or whole brain volume labels:
labels/
- freesurfer_wmparc_labels_in_hybrid_graywhite.nii.gz
- ants_labels_in_hybrid_graywhite.nii.gz
- [left,right]_cortical_surface/freesurfer_cortex_labels.vtk

Cortical surfaces with shape measures per vertex:
shapes/[left,right]_cortical_surface/
- area.vtk
- mean_curvature.vtk
- geodesic_depth.vtk
- travel_depth.vtk
- freesurfer_curvature.vtk
- freesurfer_sulc.vtk
- freesurfer_thickness.vtk

Tables of volumetric shapes per label, surface shapes per vertex, and surface shape statistics per label, sulcus, or fundus:
tables/
- volume_per_freesurfer_label.csv
- volumes_per_ants_label.csv
- thickinthehead_per_freesurfer_cortex_label.csv
- Thickinthehead_per_ants_cortex_label.csv
- [left,right]_cortical_surface/
  - label_shapes.csv
  - sulcus_shapes.csv
  - fundus_shapes.csv
  - vertices.csv
