# Supplementary figures and images for: Mindboggling morphometry of human brains
Source: PLoS Comput Biol. 2017 Feb 23;13(2):e1005350. doi: 10.1371/journal.pcbi.1005350 (PMC5322885; doi:10.1371/journal.pcbi.1005350)

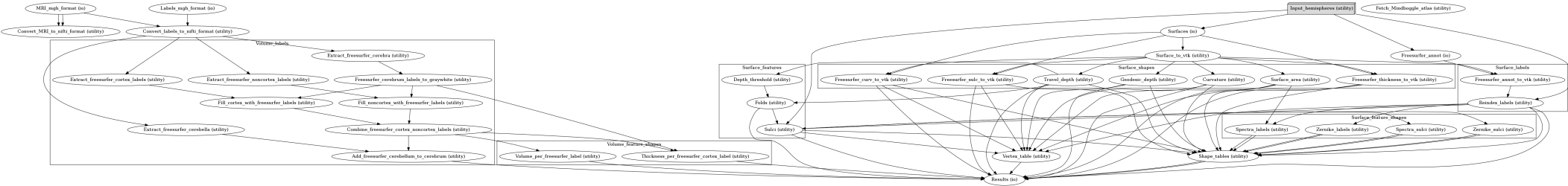

Supplement: S2 Supplement — Nipype automatically generates a flow diagram of the processing steps when running Mindboggle. (PDF) [file pcbi.1005350.s002.pdf]
