# Supplementary material for: Mindboggling morphometry of human brains
Source: PLoS Comput Biol. 2017 Feb 23;13(2):e1005350. doi: 10.1371/journal.pcbi.1005350 (PMC5322885; doi:10.1371/journal.pcbi.1005350)
Supplement: S3 Supplement — (PDF) [file pcbi.1005350.s003.pdf]

S3 Appendix: Travel depth

Joachim Giard and Arno Klein
Mindboggle supplement (http://mindboggle.info)

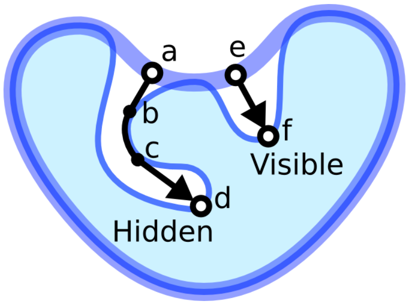

Fig A. Schematic diagram illustrating how travel depth is measured for a hidden point **d** and a visible point **f** from an outer reference surface such as the convex hull (points **a** and **e**).

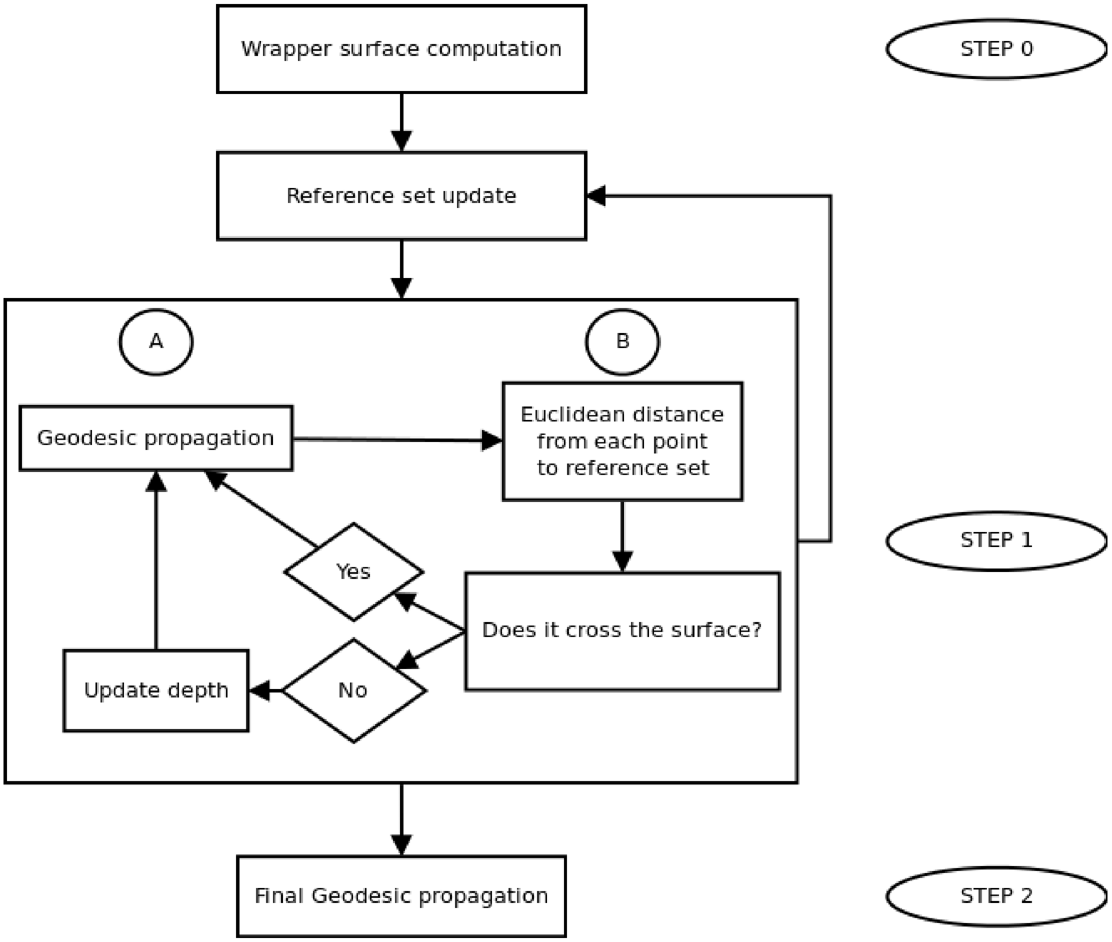

Fig B. Flowchart of the steps to compute travel depth.
The travel depth algorithm constructs a combination of Euclidean paths outside the cortical surface and estimated geodesic paths along the cortical surface. The travel depth for a given point on the surface is the minimum distance along such paths to a reference surface in three steps. An initialization step generates a mesh representation of the reference surface (Step 0). The reference surface is the convex hull for proteins and a wrapper surface for brains, and constitutes the reference set of vertices for the first iteration. Step 1 is iterated until the termination criterion is satisfied. In this step, the depth is calculated from vertices which were already visited, following a geodesic path to a close neighborhood (Step 1A) or a straight line if this line lies totally outside of the volume. At each iteration of Step 1, the newly calculated vertices act as a new set of reference vertices that replaces the wrapper surface. Step 3 is a geodesic propagation of the computed depths over the whole surface mesh.

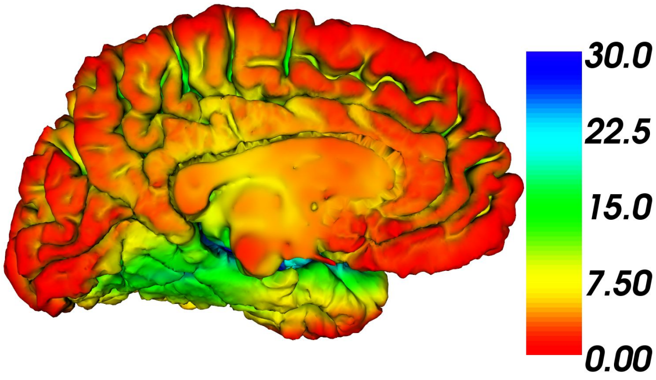

Fig C. Example travel depth measured from the convex hull. This depth map was computed on a surface mesh of a left cerebral cortex (medial view). It is clear from the positive depth (green) of the medial and inferior portions of the temporal lobe that the convex hull will lead to an exaggeration of depth in these regions.

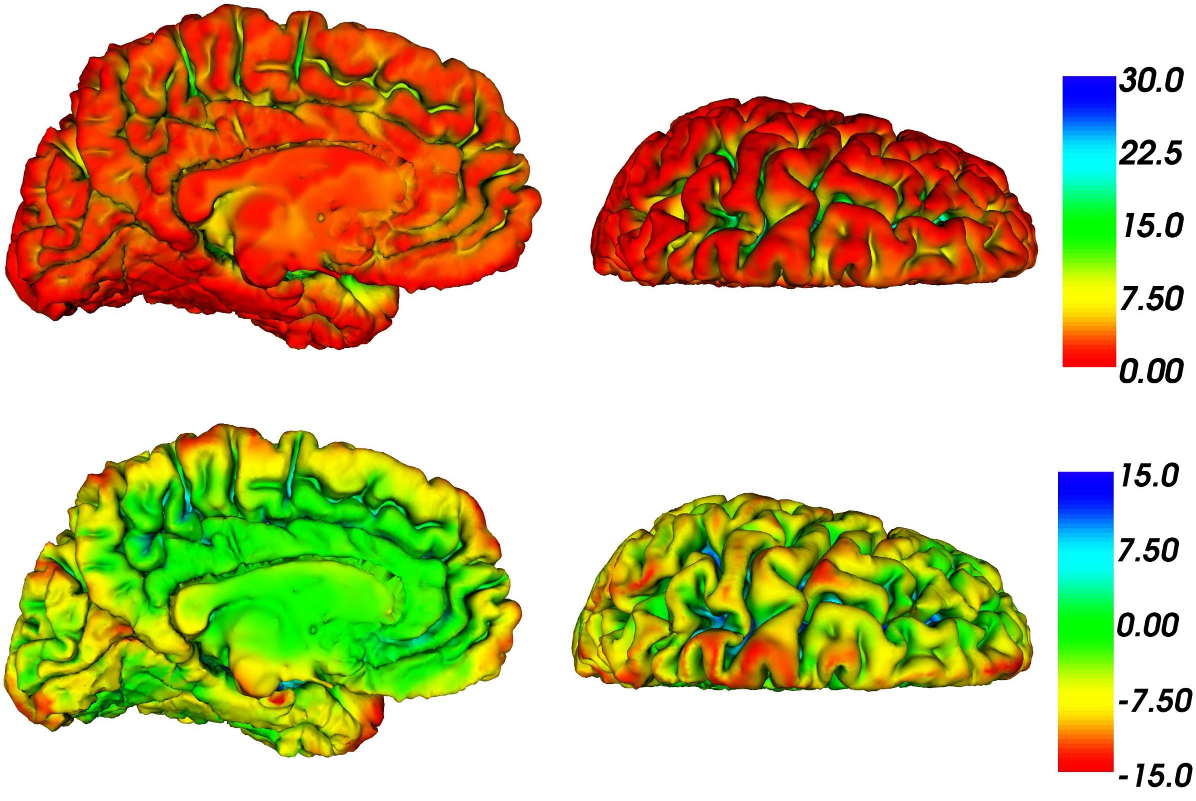

Fig D. Travel depth (top) measured from a wrapper surface (as opposed to the convex hull in Fig C) vs. FreeSurfer convexity (bottom). The left and right columns show the medial and top views of the left cerebral cortex. (Note: The colors assigned to travel depth and convexity are different because the convexity values extend below zero.)

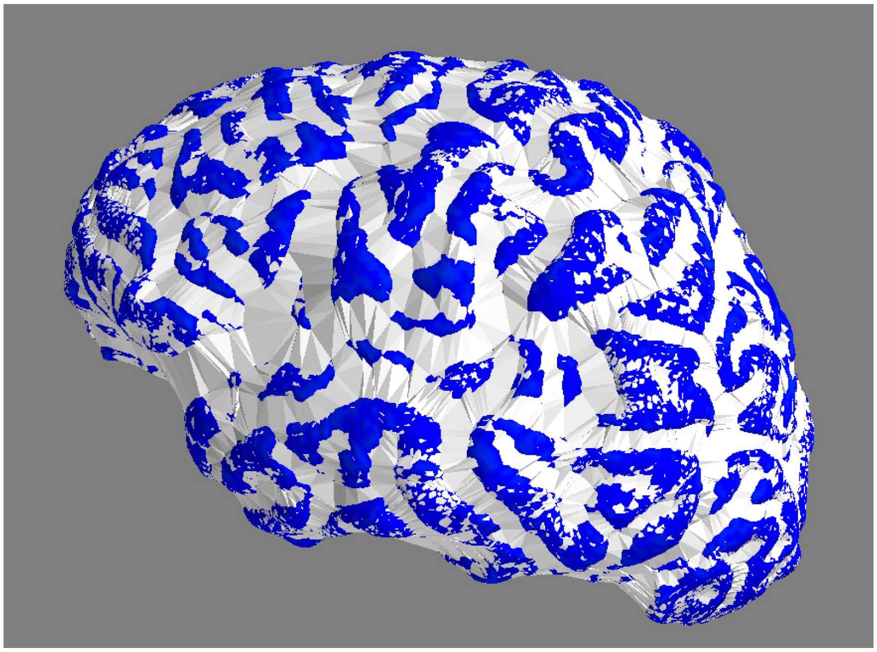

Fig E. Travel depth wrapper surface.

Table A. Statistics on travel depth and FreeSurfer convexity for a left cortical surface mesh.

	Travel depth (SD)	Convexity (SD)
Average value across vertices	6.85 mm (0.28)	0.00 mm (0.00)
Vertices with value greater than average	57.10% (0.42)	52.38% (0.30)
Vertices close to wrapper with small value	100% (0)	6.89% (0.33)
Vertices with small value close to wrapper	100% (0)	97.71% (0.91)
