# Supplementary material for: Mindboggling morphometry of human brains
Source: PLoS Comput Biol. 2017 Feb 23;13(2):e1005350. doi: 10.1371/journal.pcbi.1005350 (PMC5322885; doi:10.1371/journal.pcbi.1005350)
Supplement: S4 Supplement — (PDF) [file pcbi.1005350.s004.pdf]

S4 Appendix:
Tables of shape differences between scans and between hemispheres

Arno Klein
Mindboggle supplement (http://mindboggle.info)

Cortical region	volume	thick	area	travel	geodesic	curv	FScurv	FSthick
caudal anterior cingulate	0.04	0.05	0.05	0.10	0.10	0.06	0.18	0.04
caudal middle frontal	0.04	0.05	0.05	0.03	0.03	0.05	0.07	0.04
cuneus	0.04	0.06	0.04	0.06	0.05	0.03	0.05	0.05
entorhinal	0.21	0.06	0.09	0.20	0.16	0.06	0.14	0.05
fusiform	0.03	0.04	0.03	0.04	0.05	0.03	0.05	0.03
inferior parietal	0.03	0.04	0.02	0.03	0.02	0.04	0.05	0.04
inferior temporal	0.05	0.04	0.03	0.07	0.06	0.03	0.06	0.02
isthmus cingulate	0.04	0.08	0.07	0.11	0.13	0.04	0.11	0.04
lateral occipital	0.03	0.02	0.03	0.08	0.07	0.03	0.04	0.04
lateral orbitofrontal	0.09	0.05	0.04	0.08	0.10	0.05	0.09	0.04
lingual	0.03	0.04	0.04	0.05	0.06	0.03	0.04	0.04
medial orbitofrontal	0.07	0.06	0.06	0.14	0.10	0.06	0.11	0.05
middle temporal	0.03	0.05	0.03	0.04	0.03	0.03	0.05	0.03
parahippocampal	0.04	0.04	0.04	0.11	0.12	0.06	0.09	0.03
paracentral	0.07	0.04	0.05	0.08	0.08	0.04	0.07	0.06
pars opercularis	0.04	0.06	0.04	0.03	0.03	0.03	0.06	0.03
pars orbitalis	0.06	0.05	0.04	0.06	0.07	0.05	0.09	0.04
pars triangularis	0.03	0.06	0.03	0.06	0.05	0.04	0.09	0.04
pericalcarine	0.06	0.06	0.04	0.03	0.03	0.10	0.34	0.07
postcentral	0.04	0.02	0.03	0.03	0.03	0.03	0.05	0.06
posterior cingulate	0.04	0.08	0.05	0.09	0.09	0.05	0.09	0.04
precentral	0.04	0.03	0.02	0.02	0.02	0.03	0.04	0.04
precuneus	0.03	0.05	0.03	0.03	0.04	0.03	0.07	0.04
rostral anterior cingulate	0.04	0.09	0.06	0.10	0.10	0.08	0.23	0.05
rostral middle frontal	0.04	0.07	0.03	0.04	0.03	0.05	0.06	0.04
superior frontal	0.02	0.04	0.02	0.06	0.04	0.03	0.04	0.03
superior parietal	0.04	0.03	0.02	0.03	0.03	0.04	0.05	0.05
superior temporal	0.04	0.05	0.03	0.02	0.03	0.02	0.04	0.02
supramarginal	0.04	0.04	0.03	0.04	0.04	0.04	0.07	0.04
transverse temporal	0.06	0.07	0.04	0.02	0.03	0.04	0.07	0.06
insula	0.03	0.09	0.03	0.01	0.01	0.05	0.28	0.03

Table A. Shape differences between MRI scans

This table lists shape differences between two scans of the same brain averaged across 41 brains. The shape differences are computed for each of the 31 left cortical regions as the absolute value of the difference between the region's shape values between the two scans divided by the first scan's shape value. For the surface-based shape values, we used the median value for all vertices within each region. [thick = `thickinthehead` cortical thickness; travel = travel depth; geodesic = geodesic depth; curv = mean curvature; FScurv = FreeSurfer's curvature; FSthick = FreeSurfer's thickness]

Cortical regions	volume	thick	area	travel	geodesic	curv	FScurv	FSthick
caudal anterior cingulate	0.26	0.06	0.32	0.27	0.21	0.11	0.32	0.06
caudal middle frontal	0.14	0.04	0.20	0.14	0.13	0.10	0.16	inf
cuneus	0.12	0.06	0.17	0.32	0.24	0.10	0.15	0.04
entorhinal	0.17	0.08	0.23	0.41	0.30	0.10	0.21	0.09
fusiform	0.08	0.05	0.15	0.14	0.13	0.07	0.10	0.04
inferior parietal	0.23	0.05	0.25	0.11	0.11	0.06	0.09	0.03
inferior temporal	0.09	0.04	0.14	0.21	0.16	0.07	0.08	0.05
isthmus cingulate	0.14	0.04	0.17	0.21	0.20	0.10	0.18	0.07
lateral occipital	0.09	0.04	0.14	0.22	0.18	0.05	0.07	0.04
lateral orbitofrontal	0.06	0.05	0.11	0.23	0.26	0.09	0.12	0.05
lingual	0.11	0.04	0.12	0.15	0.13	0.08	0.11	0.05
medial orbitofrontal	0.08	0.05	0.18	0.27	0.19	0.10	0.17	0.06
middle temporal	0.11	0.04	0.12	0.17	0.15	0.07	0.10	0.05
parahippocampal	0.11	0.05	0.13	0.36	0.29	0.15	0.19	0.08
paracentral	0.16	0.04	0.18	0.25	0.20	0.08	0.16	0.04
pars opercularis	0.18	0.04	0.33	0.20	0.23	0.10	0.17	0.05
pars orbitalis	0.22	0.05	0.32	0.37	0.40	0.13	0.20	0.06
pars triangularis	0.20	0.05	0.32	0.30	0.24	0.12	0.21	0.06
pericalcarine	0.18	0.06	0.21	0.10	0.11	0.45	inf	0.06
postcentral	0.09	0.03	0.12	0.13	0.13	0.10	0.14	0.05
posterior cingulate	0.13	0.04	0.13	0.21	0.18	0.09	0.15	0.05
precentral	0.07	0.03	0.09	0.10	0.10	0.07	0.11	0.04
precuneus	0.06	0.03	0.11	0.17	0.14	0.07	0.13	0.03
rostral anterior cingulate	0.22	0.07	0.36	0.21	0.18	0.10	1.23	0.06
rostral middle frontal	0.08	0.03	0.17	0.14	0.12	0.08	0.09	0.04
superior frontal	0.06	0.03	0.14	0.19	0.14	0.06	0.07	0.04
superior parietal	0.07	0.02	0.15	0.12	0.12	0.07	0.12	0.03
superior temporal	0.07	0.03	0.12	0.10	0.10	0.09	0.11	0.04
supramarginal	0.12	0.03	0.19	0.17	0.16	0.08	0.13	0.04
transverse temporal	0.24	0.06	0.24	0.10	0.10	0.18	0.27	0.06
insula	0.06	0.04	0.07	0.05	0.03	0.10	0.32	0.05

Table B. Shape differences between left and right hemispheres
Shape differences between hemispheres are computed for each of the 31 cortical regions in all 101 of the Mindboggle-101 subjects as the absolute value of the difference between the region's left and right shape values divided by the left shape value. For the surface-based shape values, we used the median value for all vertices within each region. (Refer to **Table A** caption for

abbreviations.)
