# Supplementary material for: Mindboggling morphometry of human brains
Source: PLoS Comput Biol. 2017 Feb 23;13(2):e1005350. doi: 10.1371/journal.pcbi.1005350 (PMC5322885; doi:10.1371/journal.pcbi.1005350)
Supplement: S5 Supplement — (PDF) [file pcbi.1005350.s005.pdf]

S5 Appendix: Variance components analysis of the shapes of 62 cortical regions in 101 human brains

Elias Chaibub Neto* and Arno Klein
Sage Bionetworks, Seattle, Washington, USA
∗ E-mail: elias.chaibub.neto@sagebase.org

For more detailed plots, including summary statistical heatmaps and variation plots for shape measures computed on manually labeled cortical regions and sulci, please see:
Neto, EC, and Klein, A. (2014). shape_variation_Mindboggle101_2014.
Open Science Framework. osf.io/gzshf

Methods

Data structure

The data consist of repeated measurements on 5 distinct real-valued shape measures computed by Mindboggle (http://mindboggle.info) for each of 31 distinct regions per brain hemisphere in each of the 101 subjects. Each subject was scanned at one of 5 different laboratories. The data were organized in a nested fashion: brain hemisphere (side) is nested within subject, and subject is nested within laboratory (lab). In addition to the 5 shape measurements (`mean curvature`, `travel depth`, `geodesic depth`, `FreeSurfer convexity`, and `FreeSurfer thickness`) and the 3 nested classification factors (lab, subject, and hemisphere), the data also include 3 covariates: (i) `sex`, composed of 2 levels (`M` ≡ male, `F` ≡ female); (ii) `handedness`, composed of 2 levels (`L` ≡ left-handed, `R` ≡ right-handed) after we re-labeled 2 ambidextrous subjects as left-handed; and (iii) `age`, an integer variable.

Statistical modeling

Given the grouped nature of the data, we used linear mixed models for the statistical modeling of the data. To assess the importance of each of the covariates and nested classification factors, we fitted 24 distinct models for each shape measure and brain region combination. Tables 1 and 2 present, respectively, the 24 models and the corresponding dictionary of model terms. Table 3 presents the same 24 models expressed in R notation/syntax.

Models 1 to 8 have only fixed effects and ignore the grouping structure of the data. These simple models were used as baseline models for comparison with more complex models including nested classification factors to account for the grouping structure in the data.

Models 9 to 16 are nested mixed effect models with `sex`, `handedness`, and `age` modeled as fixed effects and `subject` and `side` (within `subject`) random effects. Note that the variance components estimates $\hat{\sigma}^2_{subj}$, $\hat{\sigma}^2_{side}$ and $\hat{\sigma}^2$ provide the following decomposition of the total variance of the shape measure:

$$\begin{aligned} \text{total variance} &= \text{variance between subjects} \\ &+ \text{variance between brain sides within a subject} \\ &+ \text{variance within brain sides} \end{aligned}$$

Models 17 to 24 include the extra "`subject` within `lab`" nesting level. For these models, the variance components estimates $\hat{\sigma}^2_{lab}$, $\hat{\sigma}^2_{subj}$, $\hat{\sigma}^2_{side}$ and $\hat{\sigma}^2$ provide a decomposition of the total variance of the shape measure into:

$$\begin{aligned}\text{total variance} = \ & \text{variance between labs} \\ & + \text{variance between subjects within a lab} \\ & + \text{variance between brain sides within a subject} \\ & + \text{variance within brain sides}\end{aligned}$$

model 1:	$y_r = \mu + \epsilon_r$
model 2:	$y_{ir} = \mu + \alpha_i + \epsilon_{ir}$
model 3:	$y_{jr} = \mu + \gamma_j + \epsilon_{jr}$
model 4:	$y_r = \mu + \beta\, x_r + \epsilon_r$
model 5:	$y_{ijr} = \mu + \alpha_i + \gamma_j + (\alpha\gamma)_{ij} + \epsilon_{ijr}$
model 6:	$y_{ir} = \mu + \alpha_i + \beta_i\, x_{ir} + \epsilon_{ir}$
model 7:	$y_{jr} = \mu + \gamma_j + \beta_j\, x_{jr} + \epsilon_{jr}$
model 8:	$y_{ijr} = \mu + \alpha_i + \gamma_j + (\alpha\gamma)_{ij} + \beta_{ij}\, x_{ijr} + \epsilon_{ijr}$
	$\epsilon_. \sim \mathrm{N}(0\,, \sigma^2)$
model 9:	$y_{klr} = \mu + b_k + b_{kl} + \epsilon_{klr}$
model 10:	$y_{iklr} = \mu + \alpha_i + b_k + b_{kl} + \epsilon_{iklr}$
model 11:	$y_{jklr} = \mu + \gamma_j + b_k + b_{kl} + \epsilon_{jklr}$
model 12:	$y_{klr} = \mu + \beta\, x_{klr} + b_k + b_{kl} + \epsilon_{klr}$
model 13:	$y_{ijklr} = \mu + \alpha_i + \gamma_j + (\alpha\gamma)_{ij} + b_k + b_{kl} + \epsilon_{ijklr}$
model 14:	$y_{iklr} = \mu + \alpha_i + \beta_i\, x_{iklr} + b_k + b_{kl} + \epsilon_{iklr}$
model 15:	$y_{jklr} = \mu + \gamma_j + \beta_j\, x_{jklr} + b_k + b_{kl} + \epsilon_{jklr}$
model 16:	$y_{ijklr} = \mu + \alpha_i + \gamma_j + (\alpha\gamma)_{ij} + \beta_{ij}\, x_{ijklr} + b_k + b_{kl} + \epsilon_{ijklr}$
	$b_k \sim \mathrm{N}(0\,, \sigma^2_{subj}), \quad b_{kl} \sim \mathrm{N}(0\,, \sigma^2_{side}), \quad \epsilon_. \sim \mathrm{N}(0\,, \sigma^2)$
model 17:	$y_{klmr} = \mu + b_m + b_{mk} + b_{mkl} + \epsilon_{klmr}$
model 18:	$y_{iklmr} = \mu + \alpha_i + b_m + b_{mk} + b_{mkl} + \epsilon_{iklmr}$
model 19:	$y_{jkmlr} = \mu + \gamma_j + b_m + b_{mk} + b_{mkl} + \epsilon_{jklmr}$
model 20:	$y_{klmr} = \mu + \beta\, x_{klmr} + b_m + b_{mk} + b_{mkl} + \epsilon_{klmr}$
model 21:	$y_{ijklmr} = \mu + \alpha_i + \gamma_j + (\alpha\gamma)_{ij} + b_m + b_{mk} + b_{mkl} + \epsilon_{ijklmr}$
model 22:	$y_{iklmr} = \mu + \alpha_i + \beta_i\, x_{iklmr} + b_m + b_{mk} + b_{mkl} + \epsilon_{iklmr}$
model 23:	$y_{jklmr} = \mu + \gamma_j + \beta_j\, x_{jklmr} + b_m + b_{mk} + b_{mkl} + \epsilon_{jklmr}$
model 24:	$y_{ijklmr} = \mu + \alpha_i + \gamma_j + (\alpha\gamma)_{ij} + \beta_{ij}\, x_{ijklmr} + b_m + b_{mk} + b_{mkl} + \epsilon_{ijklmr}$
	$b_m \sim \mathrm{N}(0\,, \sigma^2_{lab}), \quad b_{mk} \sim \mathrm{N}(0\,, \sigma^2_{subj}), \quad b_{mkl} \sim \mathrm{N}(0\,, \sigma^2_{side}), \quad \epsilon_. \sim \mathrm{N}(0\,, \sigma^2)$

Table 1. Linear models.

$y_{\cdot}$:	shape measure
$x_{\cdot}$:	age (integer valued)
α_i:	sex effect ($i = 1, 2$, $1 \equiv$ female, $2 \equiv$ male)
γ_j:	handedness effect ($j = 1, 2$, $1 \equiv$ left-handed, $2 \equiv$ right-handed)
$(\alpha\gamma)_{ij}$:	sex by handedness interaction
$\beta_{\cdot}$:	age effect
b_k:	subject random effect, $k = 1, \ldots, 101$
b_{kl}:	side within subject random effect, $l = 1, 2$ ($1 \equiv$ left-surface, $2 \equiv$ right-surface)
b_m:	lab random effect, $m = 1, \ldots, 5$
b_{mk}:	subject within lab random effect
b_{mkl}:	side within subject (within lab) random effect
$\epsilon_{\cdot}$:	residual error term

Table 2. Dictionary of model terms for Table 1.

model 1:	`measure ~ 1`
model 2:	`measure ~ sex`
model 3:	`measure ~ handedness`
model 4:	`measure ~ age`
model 5:	`measure ~ sex * handedness`
model 6:	`measure ~ sex * age`
model 7:	`measure ~ handedness * age`
model 8:	`measure ~ sex * handedness * age`
model 9:	`measure ~ 1 + (1 | subject/side)`
model 10:	`measure ~ sex + (1 | subject/side)`
model 11:	`measure ~ handedness + (1 | subject/side)`
model 12:	`measure ~ age + (1 | subject/side)`
model 13:	`measure ~ sex * handedness + (1 | subject/side)`
model 14:	`measure ~ sex * age + (1 | subject/side)`
model 15:	`measure ~ handedness * age + (1 | subject/side)`
model 16:	`measure ~ sex * handedness * age + (1 | subject/side)`
model 17:	`measure ~ 1 + (1 | lab/subject/side)`
model 18:	`measure ~ sex + (1 | lab/subject/side)`
model 19:	`measure ~ handedness + (1 | lab/subject/side)`
model 20:	`measure ~ age + (1 | lab/subject/side)`
model 21:	`measure ~ sex * handedness + (1 | lab/subject/side)`
model 22:	`measure ~ sex * age + (1 | lab/subject/side)`
model 23:	`measure ~ handedness * age + (1 | lab/subject/side)`
model 24:	`measure ~ sex * handedness * age + (1 | lab/subject/side)`

Table 3. Same linear models in Table 1 represented in R notation/syntax.

For each shape measure and brain region combination, we used the BIC score to select the best model among these 24 competing models. A BIC (Bayesian Information Criterion) score is a goodness of fit measure used to perform model selection among models with different dimensions (i.e., different number of parameters). The BIC score is proportional to the negative log likelihood of the model penalized by the number of parameters in the model. It strikes a balance between model fit (measured by the log-likelihood score) and model complexity (measured by the number of parameters in the model). In the context of linear models, an over-parameterized model will always have a larger log-likelihood score than a more parsimonious model, but it will also likely overfit the data. Nonetheless, by including a penalty proportional to the number of parameters in the model the BIC score can be used to compare models with different dimensions since the over-parameterized models are penalized to a greater extent. The smaller the BIC score, the better the model fits the data.

Results

Models 15 and 23 were the best models for the `mean curvature`, `travel depth`, `FreeSurfer convexity`, and `FreeSurfer thickness` shape measures across the 31 brain regions. Both models include `handedness` and `age` as fixed effects. They only differ by the inclusion of the extra "`subject` within `lab`" nesting level in model 23. Therefore, in the following figures we present the mean and variance component estimates for model 15 for these four shape measures. Model 9 tended to be ranked first across most brain regions (followed by model 17) for the `geodesic depth` shape measure, so we present the estimates derived from model 9 for `geodesic depth`.

Figure 1 presents an overview of the variance components decomposition results. For all shape measures and brain regions, the bulk of the variability was concentrated in the residual component (note that the results are plotted in a $\log_{10}$ scale). *For a more detailed picture of a given shape measure and brain region you can inspect the corresponding variation plot (osf.io/gzshf).*

Figure 2 presents the estimates of the population means derived from the mixed effects models. Note that for `geodesic depth` we have a single column (overall.mu) which corresponds to the estimate of the μ parameter in model 9. For all other shape measures we have 2 columns (left.handed.mu, right.handed.mu) which correspond, respectively, to the estimates of $\mu + \gamma_1$ and $\mu + \gamma_2$ parameters in model 15 (that is, the population means for left-handed and right-handed people, respectively).

Tables 1 to 5 present the data used in the generation of these figures. Tables 6 to 9 present BIC scores for four of the shape measures.

Figures

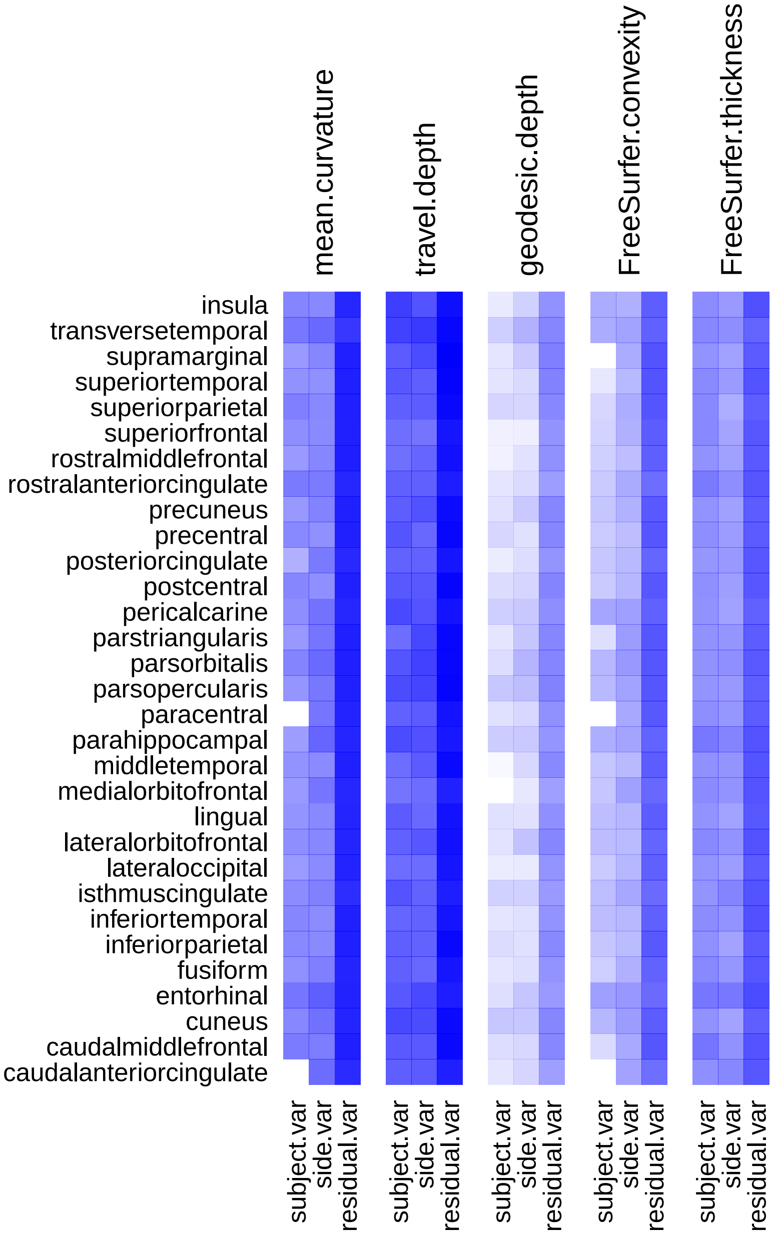

Figure 1. Variance components estimates in $\log_{10}$ scale.

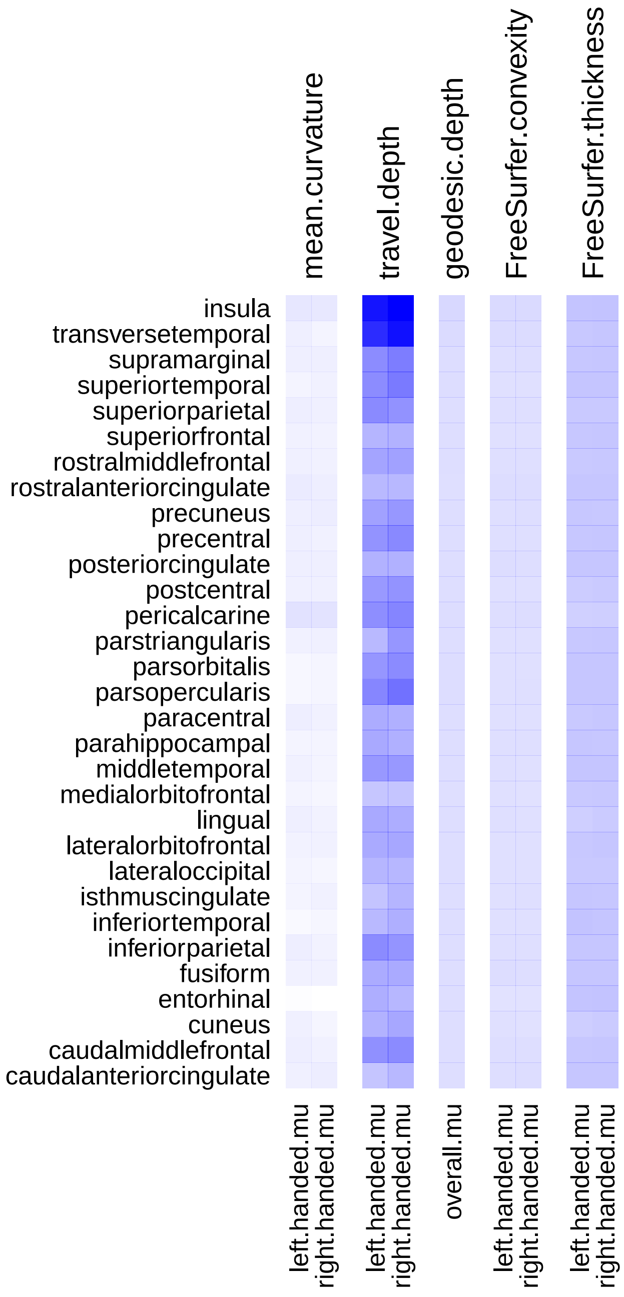

Figure 2. Mean estimates.

Tables

Table 1: Mean curvature mean and variance values per region

	mu_L	mu_R	sig2_subj	sig2_side	sig2
caudalanteriorcingulate: 2	-1.9646759	-1.6133236	0.000000000	0.11911361	5.637640
caudalmiddlefrontal: 3	-1.6939180	-2.0584152	0.047309070	0.04126164	11.673396
cuneus: 5	-1.9004044	-2.4063073	0.023036867	0.08529971	8.223499
entorhinal: 6	-3.3538056	-3.6821572	0.071264116	0.26433676	8.932195
fusiform: 7	-2.0206615	-2.1021870	0.013778937	0.03698830	8.328005
inferiorparietal: 8	-1.7879809	-2.1505798	0.021603439	0.01871475	11.584457
inferiortemporal: 9	-2.9024409	-2.4853642	0.025988620	0.01718070	10.269853
isthmuscingulate: 10	-2.3052108	-2.0530746	0.016935931	0.03475560	5.069706
lateraloccipital: 11	-2.3293248	-2.4735612	0.007083792	0.01782347	8.635402
lateralorbitofrontal: 12	-2.1530630	-2.1100545	0.015932655	0.02256718	7.945797
lingual: 13	-1.8660691	-2.1481973	0.010326448	0.02142574	7.951898
medialorbitofrontal: 14	-2.2619382	-2.5459289	0.007769905	0.06992965	7.261699
middletemporal: 15	-2.0636435	-2.2855140	0.011711308	0.01824557	10.937725
parahippocampal: 16	-2.3290528	-2.2976986	0.006315487	0.15737938	7.617145
paracentral: 17	-1.7413352	-2.1053616	0.000000000	0.07143120	8.443684
parsopercularis: 18	-2.5784662	-2.4002403	0.009878078	0.05879899	11.198582
parsorbitalis: 19	-2.6498301	-2.4368318	0.028455308	0.13310886	11.540800
parstriangularis: 20	-2.0630806	-1.9927985	0.008045435	0.06898698	11.845844
pericalcarine: 21	-0.3627639	-0.4734272	0.015745534	0.07658601	7.218891
postcentral: 22	-1.9334892	-1.9197625	0.023211111	0.01508505	10.290260
posteriorcingulate: 23	-1.8144612	-1.8325023	0.001976674	0.04737118	6.713240
precentral: 24	-1.8095272	-2.0533020	0.019708800	0.01425260	11.074243
precuneus: 25	-1.8450386	-1.6291366	0.008104536	0.03044350	10.219587
rostralanteriorcingulate: 26	-1.3037218	-1.6773242	0.049823919	0.04536554	7.100370
rostralmiddlefrontal: 27	-1.9045841	-2.0901363	0.007940850	0.02426930	12.306761
superiorfrontal: 28	-2.1057824	-2.2150371	0.015505640	0.01910455	10.370381
superiorparietal: 29	-1.7866883	-1.9290479	0.035581720	0.01956873	11.749346
superiortemporal: 30	-2.3379290	-2.0486796	0.012312071	0.01418901	11.393688
supramarginal: 31	-1.8534484	-1.8222733	0.007644038	0.02335495	11.681114
transversetemporal: 34	-1.8348014	-2.3305493	0.058914890	0.14041556	2.763680
insula: 35	-0.8940259	-0.9301153	0.027015605	0.01969170	7.350533

Table 2: Travel depth mean and variance values per region

	mu_L	mu_R	sig2_subj	sig2_side	sig2
caudalanteriorcingulate: 2	2.908827	4.390086	0.26273312	0.32216772	11.166426
caudalmiddlefrontal: 3	8.822163	9.321539	0.34371635	0.35037770	41.476752
cuneus: 5	4.939416	6.263497	0.93816728	0.74787219	38.870542
entorhinal: 6	5.364478	4.552021	0.36538268	0.90667992	13.159925
fusiform: 7	5.876810	5.952691	0.23411494	0.14745465	20.012670
inferiorparietal: 8	9.295072	8.337746	0.26474506	0.20183172	41.811629
inferiortemporal: 9	4.183588	5.331245	0.16666735	0.20352165	21.510881
isthmuscingulate: 10	3.022776	4.677700	0.52506112	0.20787633	12.006928
lateraloccipital: 11	4.647892	4.555075	0.11287421	0.12218326	20.127287
lateralorbitofrontal: 12	5.978216	6.316145	0.21471711	0.39148900	26.575029
lingual: 13	6.085052	5.648819	0.32105738	0.14102661	25.839482
medialorbitofrontal: 14	2.784618	2.992110	0.07467968	0.11202420	9.624075
middletemporal: 15	7.800564	7.855389	0.10696417	0.33047961	39.614431
parahippocampal: 16	6.029435	5.199095	0.76328940	0.57625415	16.390874
paracentral: 17	5.693255	5.220015	0.20942002	0.32854616	23.648017
parsopercularis: 18	9.835592	12.244041	0.77089314	1.19907867	59.457262
parsorbitalis: 19	8.052297	9.313831	0.42789908	1.57559078	49.665636
parstriangularis: 20	4.325956	8.035255	0.11369699	1.08169516	48.826880
pericalcarine: 21	9.027955	10.051042	0.90535113	0.54249963	24.059514
postcentral: 22	7.689919	8.539968	0.36657728	0.36906470	56.107320
posteriorcingulate: 23	4.943409	5.034161	0.20527867	0.20318742	19.305122
precentral: 24	8.533288	9.499526	0.43347605	0.15283913	49.320424
precuneus: 25	6.857130	7.967200	0.28162255	0.54277301	36.888115
rostralanteriorcingulate: 26	4.272170	4.433297	0.24899008	0.23633339	13.011533
rostralmiddlefrontal: 27	6.493383	6.794857	0.08813629	0.16354173	27.046853
superiorfrontal: 28	4.772673	4.970345	0.09316461	0.07220226	20.145720
superiorparietal: 29	9.410513	8.574138	0.26077990	0.31168172	43.602626
superiortemporal: 30	9.175236	11.322221	0.38526973	0.26043419	61.570555
supramarginal: 31	9.221037	10.885826	0.33357991	0.79940538	71.311463
transversetemporal: 34	20.024956	23.075414	1.51919779	2.32137437	44.712003
insula: 35	22.497879	24.832637	1.76948715	0.54084168	29.289498

Table 3: Geodesic depth mean and variance values per region

	mu	sig2_subj	sig2_side	sig2
caudalanteriorcingulate: 2	0.10876145	8.540137e-05	2.155707e-04	0.005735945
caudalmiddlefrontal: 3	0.20672329	1.405328e-04	1.916866e-04	0.022901589
cuneus: 5	0.16442286	5.096960e-04	5.015735e-04	0.023892951
entorhinal: 6	0.11356239	1.359486e-04	6.071509e-04	0.007505954
fusiform: 7	0.14956220	8.285491e-05	1.341376e-04	0.010899991
inferiorparietal: 8	0.19035957	1.540057e-04	1.194686e-04	0.020971043
inferiortemporal: 9	0.13032484	8.443557e-05	1.175144e-04	0.011829854
isthmuscingulate: 10	0.13021684	2.913291e-04	2.894364e-04	0.007531595
lateraloccipital: 11	0.10758544	6.034204e-05	6.777100e-05	0.010589475
lateralorbitofrontal: 12	0.18916881	9.206473e-05	7.286072e-04	0.023227473
lingual: 13	0.14397953	1.143569e-04	1.077340e-04	0.014926456
medialorbitofrontal: 14	0.07873026	1.731992e-05	7.859723e-05	0.005881223
middletemporal: 15	0.17461209	2.952878e-05	1.818783e-04	0.019642103
parahippocampal: 16	0.13303999	3.732840e-04	4.478648e-04	0.009880747
paracentral: 17	0.13349202	1.134478e-04	1.929274e-04	0.013616939
parsopercularis: 18	0.28611134	5.104992e-04	8.448066e-04	0.031570206
parsorbitalis: 19	0.21913221	1.422498e-04	1.627297e-03	0.027178661
parstriangularis: 20	0.18745774	8.140182e-05	5.725029e-04	0.024734192
pericalcarine: 21	0.24349602	3.551833e-04	4.585307e-04	0.015534629
postcentral: 22	0.19885442	1.500128e-04	2.160770e-04	0.028445429
posteriorcingulate: 23	0.12946234	5.956961e-05	1.454169e-04	0.010279177
precentral: 24	0.21356886	2.106062e-04	1.100537e-04	0.025863662
precuneus: 25	0.19837843	1.040346e-04	4.382145e-04	0.023298807
rostralanteriorcingulate: 26	0.10429126	8.291946e-05	1.574992e-04	0.006600894
rostralmiddlefrontal: 27	0.15173198	4.120515e-05	9.756043e-05	0.013571889
superiorfrontal: 28	0.11807782	4.337756e-05	4.916746e-05	0.010730130
superiorparietal: 29	0.19886998	2.129506e-04	2.047426e-04	0.022850541
superiortemporal: 30	0.25845599	8.740657e-05	1.749888e-04	0.032775807
supramarginal: 31	0.24143904	8.341553e-05	4.195554e-04	0.035882348
transversetemporal: 34	0.53976151	3.259074e-04	1.831153e-03	0.024633241
insula: 35	0.75147945	6.943335e-05	2.928809e-04	0.013522412

Table 4: FreeSurfer convexity mean and variance values per region

	mu_L	mu_R	sig2_subj	sig2_side	sig2
caudalanteriorcingulate: 2	0.118733778	0.218756373	0.0000000000	0.0041627352	0.09847667
caudalmiddlefrontal: 3	0.142603415	0.128505096	0.0001723431	0.0030792896	0.40823457
cuneus: 5	-0.188217849	-0.236007441	0.0013592559	0.0068444630	0.31641205
entorhinal: 6	-0.367327588	-0.394087214	0.0057705233	0.0087361982	0.13619453
fusiform: 7	0.227530817	0.158152913	0.0003473750	0.0019095904	0.20416742
inferiorparietal: 8	0.073579236	0.030316008	0.0005279175	0.0010115500	0.37297250
inferiortemporal: 9	-0.227300932	-0.093562614	0.0009544730	0.0012756580	0.24333934
isthmuscingulate: 10	-0.132946172	0.008820128	0.0009107362	0.0035642893	0.13513821
lateraloccipital: 11	-0.204201120	-0.174206284	0.0004061473	0.0013482482	0.24066212
lateralorbitofrontal: 12	-0.054651898	-0.110014616	0.0009769749	0.0011070491	0.18205332
lingual: 13	0.027225061	-0.018170522	0.0009061470	0.0014122934	0.30229361
medialorbitofrontal: 14	-0.212249233	-0.336624222	0.0005795722	0.0054150929	0.15344121
middletemporal: 15	0.071628421	-0.010907508	0.0005982494	0.0011834910	0.30967070
parahippocampal: 16	0.087491792	0.035948116	0.0022616907	0.0039126025	0.19128481
paracentral: 17	-0.057810428	-0.062114784	0.0000000000	0.0033245646	0.36084282
parsopercularis: 18	-0.035535797	0.031557438	0.0012213810	0.0044987884	0.41124968
parsorbitalis: 19	-0.186196642	-0.110181442	0.0013585418	0.0081325988	0.41417753
parstriangularis: 20	-0.188932229	-0.026018515	0.0001323680	0.0069461003	0.41780549
pericalcarine: 21	0.171297267	0.255125384	0.0038692211	0.0056984023	0.22148795
postcentral: 22	-0.068708047	-0.062727424	0.0004219237	0.0013778157	0.38872078
posteriorcingulate: 23	0.309138606	0.208582691	0.0004956055	0.0012019565	0.16562237
precentral: 24	0.035019155	0.036391492	0.0003900327	0.0008286903	0.41969548
precuneus: 25	0.040684245	0.112356890	0.0005273178	0.0018404424	0.34969709
rostralanteriorcingulate: 26	0.263050467	0.225369214	0.0004293334	0.0029767907	0.10557882
rostralmiddlefrontal: 27	0.002363582	0.009938400	0.0003118705	0.0009170744	0.26370457
superiorfrontal: 28	-0.122509024	-0.109382465	0.0002400504	0.0020116352	0.28434453
superiorparietal: 29	0.031060955	0.009850810	0.0002025333	0.0024428874	0.42994640
superiortemporal: 30	-0.112306773	-0.030108818	0.0000783506	0.0011961732	0.45667137
supramarginal: 31	0.033636482	0.109173675	0.0000000000	0.0026713989	0.46337782
transversetemporal: 34	0.342587711	0.358172282	0.0025759434	0.0046281102	0.21290818
insula: 35	0.658690708	0.649950765	0.0026419967	0.0019128761	0.27587704

Table 5: FreeSurfer thickness mean and variance values per region

	mu_L	mu_R	sig2_subj	sig2_side	sig2
caudalanteriorcingulate: 2	2.770639	2.784046	0.014854571	0.022279403	0.3985204
caudalmiddlefrontal: 3	2.558892	2.785496	0.068478804	0.012540913	0.4790259
cuneus: 5	1.913028	2.139964	0.012665457	0.004138412	0.2603659
entorhinal: 6	3.047758	3.217990	0.065682829	0.057534832	0.8188346
fusiform: 7	2.813329	2.733994	0.020922657	0.009350653	0.3974182
inferiorparietal: 8	2.685422	2.727383	0.013692157	0.004171075	0.3384569
inferiortemporal: 9	3.115784	2.910109	0.016699047	0.011174295	0.5763199
isthmuscingulate: 10	2.858025	2.651990	0.010200465	0.028049292	0.5340505
lateraloccipital: 11	2.371334	2.352359	0.010679654	0.006590075	0.3301511
lateralorbitofrontal: 12	2.513763	2.668454	0.019478931	0.011355397	0.5512252
lingual: 13	1.846035	2.168769	0.011997320	0.004621341	0.3598591
medialorbitofrontal: 14	2.397132	2.514900	0.018078920	0.011524783	0.5319622
middletemporal: 15	2.891214	2.936113	0.014027593	0.010269694	0.4321235
parahippocampal: 16	2.692659	2.653202	0.060000967	0.027818592	0.5227127
paracentral: 17	2.398629	2.645474	0.016212140	0.009225692	0.3141635
parsopercularis: 18	2.711755	2.784389	0.010271932	0.008204965	0.2504628
parsorbitalis: 19	2.830011	2.770044	0.012196042	0.015149751	0.3473796
parstriangularis: 20	2.490471	2.630728	0.012491734	0.009803283	0.3063362
pericalcarine: 21	1.725996	1.783490	0.012327533	0.004868801	0.2176258
postcentral: 22	2.091484	2.225070	0.014422305	0.006025518	0.3877497
posteriorcingulate: 23	2.782130	2.680288	0.009448248	0.008205112	0.3995141
precentral: 24	2.599958	2.778354	0.016021647	0.006697638	0.3256826
precuneus: 25	2.622358	2.549997	0.011610984	0.005536870	0.3155480
rostralanteriorcingulate: 26	2.861973	2.866584	0.049610033	0.015946495	0.4243849
rostralmiddlefrontal: 27	2.358139	2.551242	0.012440702	0.005402452	0.3439743
superiorfrontal: 28	2.623128	2.873055	0.020498676	0.004052127	0.3945112
superiorparietal: 29	2.391569	2.412446	0.020424801	0.002269392	0.2775097
superiortemporal: 30	2.990558	2.966190	0.018420617	0.007050802	0.4868802
supramarginal: 31	2.574476	2.748686	0.011547750	0.004804002	0.3235135
transversetemporal: 34	2.479728	2.710507	0.022940771	0.015621186	0.1847719
insula: 35	3.021156	3.118172	0.016891817	0.007512457	0.6309455

Table 6: Mean curvature top models and BIC scores per region

	top1	top2	top3	BIC1	BIC2	BIC3
2	15	23	16	1405671.62841	1405680.65650	1405737.88450
3	23	15	24	4100038.32650	4100062.40415	4100109.29606
5	15	23	16	3005449.44903	3005462.69462	3005507.44909
6	15	23	16	579414.99081	579423.88032	579468.81506
7	23	15	24	4180787.55423	4180803.22063	4180856.32898
8	23	15	16	7436830.32176	7436831.71403	7436892.41283
9	15	23	16	5285465.25639	5285473.55638	5285538.56939
10	15	23	16	1206062.36202	1206074.86914	1206121.53064
11	15	23	16	7671123.73143	7671134.33568	7671194.42544
12	23	15	24	4040730.35157	4040731.06853	4040781.29852
13	15	23	24	4647806.70532	4647806.82832	4647879.94487
14	15	23	16	1914090.11214	1914098.15244	1914160.18665
15	15	23	16	6419647.73983	6419660.95354	6419722.75465
16	15	23	16	968083.02936	968086.67297	968145.92115
17	15	23	16	2469727.56518	2469740.68083	2469794.36457
18	15	23	16	2428738.30015	2428749.07805	2428803.62514
19	15	23	16	1112372.87289	1112385.12969	1112435.42463
20	15	23	16	2318929.97109	2318942.95787	2318998.43938
21	15	23	16	2036791.34509	2036799.07569	2036858.18968
22	23	15	24	7170440.39145	7170449.78402	7170513.46440
23	15	23	24	1828064.95718	1828065.72079	1828131.61927
24	15	23	16	7559815.64969	7559829.83115	7559879.93021
25	15	23	16	5791398.44336	5791457.82998	5791471.62217
26	23	15	24	522213.88602	1522238.12250	1522282.55119
27	15	23	16	6918718.40945	6918727.61529	6918784.54003
28	23	15	16	12023970.92870	12023978.33195	12024047.79474
29	23	15	24	7033779.40343	7033782.46490	7033849.12479
30	15	23	16	7387359.80076	7387406.72495	7387436.36871
31	15	23	16	5703414.41702	5703421.48104	5703481.17729
34	15	23	16	524415.89703	524422.74899	524475.50554
35	15	23	16	2719704.56470	2719710.37649	2719771.66897

Table 7: Travel depth top models and BIC scores per region

	top1	top2	top3	BIC1	BIC2	BIC3
2	15	23	16	1616038.07003	1616050.70657	1616082.84289
3	15	23	16	5081706.55612	5081717.74058	5081768.93139
5	15	23	16	3949418.80977	3949430.06884	3949474.21333
6	15	23	16	624187.03028	624195.88352	624231.39879
7	15	23	16	4920265.50402	4920278.73237	4920323.12746
8	15	23	16	9242089.36772	9242097.61082	9242151.94746
9	15	23	16	6041292.26431	6041305.27249	6041356.90278
10	15	23	16	1440185.45055	1440195.79288	1440241.23852
11	23	15	24	8971129.32578	8971137.64898	8971188.64460
12	15	23	16	5034328.70975	5034341.61507	5034386.90849
13	15	23	16	5763122.63192	5763132.06231	5763181.94591
14	15	23	16	2025976.59513	2025986.27611	2026037.17759
15	15	23	16	7999435.73620	7999449.75654	7999495.15620
16	15	23	16	1120558.32892	1120570.52853	1120607.50542
17	15	23	16	2981362.82103	2981371.09526	2981416.54968
18	15	23	16	3200600.41026	3200611.20106	3200652.66607
19	15	23	16	1419628.44797	1419636.33745	1419680.82525
20	15	23	16	2937524.12494	2937534.61200	2937581.51254
21	15	23	16	2546118.62159	2546129.71963	2546166.60859
22	23	15	24	9523173.95583	9523207.40245	9523234.23348
23	15	23	16	2235288.12415	2235300.98612	2235328.87088
24	23	15	24	9713776.13790	9713793.72908	9713838.90951
25	15	23	16	7231575.70930	7231588.79287	7231637.30322
26	15	23	16	1714496.04184	1714508.70889	1714546.10200
27	15	23	16	7937522.74472	7937536.81763	7937587.96242
28	23	15	24	13566340.78166	13566350.97783	13566412.24750
29	15	23	16	8773547.66412	8773561.55858	8773612.67964
30	15	23	16	9751901.67622	9751914.00651	9751965.33389
31	15	23	16	7651806.03929	7651817.65426	7651865.39067
34	15	23	16	902541.95449	902552.54590	902587.79071
35	15	23	16	3497841.13346	3497851.29890	3497896.17261

Table 8: FreeSurfer convexity top models and BIC scores per region

	top1	top2	top3	BIC1	BIC2	BIC3
2	15	23	16	160849.40042	160862.03696	160926.89914
3	15	23	16	1504178.56256	1504191.90689	1504262.86431
5	15	23	16	1026124.46392	1026137.78124	1026194.05836
6	15	23	16	98060.00435	98071.51562	98122.92070
7	15	23	16	1054052.74098	1054066.37213	1054136.08325
8	15	23	16	2604806.17921	2604815.50687	2604891.48791
9	15	23	16	1458373.76151	1458386.01718	1458456.87421
10	15	23	16	226661.64073	226674.14785	226740.63306
11	15	23	16	2172068.81490	2172081.08940	2172151.10408
12	15	23	16	934177.15348	934189.33716	934257.63054
13	15	23	16	1554038.28269	1554051.27092	1554122.56653
14	15	23	16	383372.45149	383385.34300	383452.23297
15	15	23	16	2045071.20218	2045081.98262	2045154.19950
16	15	23	16	236027.41552	236039.61491	236103.48933
17	15	23	16	903935.48692	903948.60257	904016.41049
18	15	23	16	901713.08066	901725.47795	901784.94090
19	15	23	16	412372.97157	412383.92239	412443.26594
20	15	23	16	858801.51422	858814.50100	858880.28879
21	23	15	24	563618.44260	563622.63552	563696.70842
22	15	23	16	2626454.34578	2626463.59356	2626540.64792
23	15	23	16	401426.49573	401439.29188	401507.55196
24	15	23	16	2840749.19664	2840801.75346	2840835.72672
25	15	23	16	2005613.32736	2005627.25777	2005695.79436
26	15	23	16	187841.04404	187853.71109	187922.26725
27	15	23	16	1947549.85636	1947561.19595	1947635.80679
28	15	23	16	3671265.55541	3671271.30560	3671350.04690
29	15	23	16	2645742.28301	2645754.34761	2645827.79002
30	15	23	16	2879321.74036	2879334.34007	2879412.27097
31	15	23	16	2228414.69407	2228428.58361	2228502.54265
34	15	23	16	176073.48213	176085.30134	176147.53910
35	15	23	16	872969.70773	872982.94813	873028.72474

Table 9: FreeSurfer thickness top models and BIC scores per region

	top1	top2	top3	BIC1	BIC2	BIC3
2	15	23	16	590972.00900	590978.24907	591038.11853
3	23	15	24	1628395.46894	1628418.21253	1628467.68067
5	15	23	16	907754.88882	907766.91378	907830.77689
6	23	15	24	304562.95009	304586.60001	304622.39725
7	23	15	24	1615911.30133	1615943.70309	1615982.46765
8	15	23	16	2468633.83891	2468634.73945	2468705.85753
9	15	23	16	2339695.21941	2339705.54212	2339768.74922
10	23	15	24	598351.46259	598353.79696	598417.50499
11	15	23	16	2658006.38688	2658014.44814	2658084.86942
12	23	15	24	1845887.92002	1845897.62099	1845960.27277
13	23	15	24	1719249.24373	1719250.18258	1719324.64928
14	23	15	24	876867.36748	876870.05735	876935.41564
15	15	23	16	2454405.64620	2454407.64792	2454479.84667
16	23	15	24	436019.71514	436036.84659	436084.66897
17	23	15	16	835478.91088	835481.43235	835545.27991
18	15	23	16	672801.70348	672812.80283	672869.20808
19	15	23	16	375572.12924	375584.38604	375638.73389
20	15	23	16	723540.80473	723553.05191	723610.77949
21	15	23	16	556243.37257	556253.47878	556314.80509
22	23	15	24	2623362.28702	2623371.11746	2623440.28162
23	15	23	16	741000.36920	741002.50802	741067.95104
24	15	23	16	2475603.97245	2475612.40152	2475675.84290
25	15	23	16	1890675.47438	1890688.90804	1890748.78241
26	23	15	24	629185.51525	629221.25310	629252.24338
27	23	15	24	2291675.48439	2291683.63118	2291751.51869
28	23	15	24	4432048.80889	4432058.47871	4432125.99475
29	23	15	24	2065262.19787	2065284.38157	2065341.30492
30	15	23	16	2969552.42402	2969560.35853	2969622.45350
31	15	23	16	1841837.66063	1841843.92697	1841906.92511
34	15	23	16	157122.83662	157134.65583	157179.93557
35	15	23	24	1338553.30397	1338553.50312	1338621.23296
